# Key Terms and Concepts in Alcohol Use and Problems: A Critical Evaluation

**DOI:** 10.1177/29768357241312555

**Published:** 2025-03-12

**Authors:** James Morris, Cassandra L Boness, Micah Hartwell

**Affiliations:** 1 Department of Psychology, London South Bank University, London, UK; 2 Department of Psychology and Center on Alcohol, Substance use, And Addictions, University of New Mexico, Albuquerque, NM, USA; 3 Center for Health Sciences, Oklahoma State University, Stillwater, OK, USA

**Keywords:** Alcohol, stigma, alcohol use disorder, addiction, recovery, person first language, terminology

## Abstract

**Objective:**

Alcohol use is linked to a wide and complex range of individual and societal harms. Decisions on whether and how to address alcohol-related harms are shaped by the way in which such problems are understood, particularly through the use of language and concepts in professional and lay discourse. However, all terms and concepts have a complex set of implications which vary by context. For example, some language, particularly that associated with a historically dominant ‘alcoholism’ model, may be clearly harmful in some contexts (eg, via public stigma) and potentially valuable in others (eg, via facilitating recovery processes), or hold ‘mixed blessings’. Careful empirical attention is required to assess the implications of key terms and concepts used in efforts to understand and address alcohol use and problems amongst the public, researchers, policy makers and practitioners.

**Methods:**

We take an author-led and empirically informed approach to critically evaluate common terms and concepts to describe alcohol use and related constructs.

**Results:**

We identify how alcohol-related framing and discourse is highly relevant to alcohol-related outcomes via key issues including stigma, public health goals, political and commercial interests.

**Conclusions:**

Recommendations are made for key partners to consider in the use and evolution of key terms and concepts relating to the broad spectrum of alcohol use and problems.

## Introduction

Ethanol (ethyl alcohol) is widely available as a legal drug, despite accounting for over 3 million annual deaths.^1^ The cultural normalization of alcohol use, particularly in Western cultures such as the United States and United Kingdom, is therefore a major obstacle to evidence-based policies to reduce associated harm.^2,3^ Although alcohol consumption is clearly linked to its price and availability, it is also indirectly influenced via less tangible processes such as the *terms* and *concepts* used to describe consumption in public and professional alcohol-related discourse.^4^ As the adage goes, *language matters* because the terms and concepts used in any domain have multiple implications for how people and society understand and respond to an issue.^5-8^ For example, whether via social process mechanisms such as *stigma*, or commercial practices of industry marketing and *issue framing*, these discourses shape public attitudes towards alcohol, and in turn, the level of regulation or other actions to reduce its harms.^3,9-12^

The ways in which terms and concepts shape substance use and harms, medical treatment and prevention efforts can be seen across multiple domains. For instance, drug policy decisions (including those related to alcohol) are embedded within moralistic ideas and discourse, reflecting normative and stigma-embedded ideas about what constitutes ‘problematic’ drug use or drug ‘users’.^13-15^ The legal status and related policy responses to different drugs therefore typically reflects complex socio-historical, commercial and political factors, rather than the level of harm they cause to individuals and society.^14,16^ As such, the terms and concepts used to describe alcohol use problems have a range of complex and far-reaching implications, spanning individual level behaviours through to key population level policy controls of price, availability or marketing restrictions.^2,12,17^

Increasing attention has been given to the role of language and issue framing, particularly in the context of ‘substance use disorder’ (SUD) and ‘alcohol use disorder’ (AUD) policy, research and practice. This attention partially reflects growing evidence around the implications of certain terms and concepts, particularly in terms of their role in stigma processes as a major barrier to addressing alcohol problems.^18,19^ Notably, stigma is a social process which is enacted via *labelling* of the stigmatized group or characteristic (eg, ‘alcoholic’). Labelling serves to separate and mark as different those subjected to prejudice and discrimination, which occurs through complex social and structural means.^20-22^ Many stigma processes are thus enacted via the use and exclusion of certain terms and their embedded meanings.^23-26^ In turn, various terms and concepts relating to substance use, SUD and related issues have been subject to experimental research, debate and advocacy about language choice.

Resources aimed at guiding terminology have been produced by many major bodies. For example, the National Institute on Drug Abuse published ‘*Words Matter: Preferred Language for Talking About Addiction*’ in 2021.^27^ Further, a number of organizations, research centres and academic journals have made name changes to reduce stigmatizing language, such as replacing ‘alcoholism’ or ‘abuse’ within journal titles,^28^ published calls for authors to avoid stigmatizing terminology,^29,30^ or published positions on how they manage disputes over terminology.^31^ In 2023, legislation in the US state of New Jersey replaced ‘. . . statutory terms regarding alcohol and substance use, alcoholism, addiction, drug addict, and similar terms with the terms “substance use disorder” and “person with substance use disorder”.’^32^

Although emerging evidence highlights how certain terms are more straightforwardly problematic, at least in most contexts outside of self-labelling (eg, ‘alcoholic’), many terms and concepts are subject to ongoing discussion, reflecting the various motives or contexts in which they may be used (see Tables 1 and 2). In this manuscript we seek to identify and critically evaluate key terms and concepts relating to alcohol use and problems within professional (Table 1) and public (Table 2) discourse. Our approach is based on author consensus in determining what qualifies as ‘key terms’ as well as common professional classification systems and associated terminology (eg, *International Classification of Diseases*). Although we have separated tables by public and professional contexts, we acknowledge considerable overlap since some terms are prevalent in both contexts (eg, ‘recovery’). We seek to take an author-led, empirically-informed approach, paying particular attention to evaluations of word choice and framing studies, as well as evidence pertaining to conceptualizations of alcohol use and related issues.

**Table 1.** Key alcohol use and problem terms and concepts in professional contexts.

Term/concept	Summary	Issues and key considerations	Context dependent considerations*	Conclusions and recommendations
Alcohol dependence	Typically understood as a maladaptive pattern of drinking that results in various physiological adaptations such as compulsive use/neurobiological adaption, tolerance and withdrawal.	Although the concept of alcohol dependence still has merit, its use as a DSM diagnostic label is now largely outdated (though it still appears in ICD-11). The term captures an important but somewhat narrow conceptualization of what alcohol use disorder (AUD) might encompass (eg, it neglects important psychological components of alcohol use disorder/addiction).^33^	Alcohol dependence is likely to have greater understanding amongst the general public than AUD-related concepts whilst avoiding stigma-related concerns of other terminology (eg, alcoholism).^4^ Alcohol dependence is still used in some classification systems and may be used for professional purposes (eg, billing for the management of alcohol withdrawal in the US).	Appropriateness may depend on the classification system being used. Dependence may also have utility in focusing on physiological components in some contexts (eg, assessing and managing withdrawal as a priority)
Alcohol Use Disorder (AUD)	AUD concepts are contemporary classification attempts to capture alcohol problems for prevention, treatment, research and policy purposes.	Different AUD classifications vary in the way they capture and classify alcohol problems.^4^ Notably, DSM-5 captures AUD as broadly a problem of impaired control over use, social impairment, risky use and physiological adaptations (ie, dependence), whereas the UK’s NICE^34^ and WHO’s AUDIT approach have taken a broader continuum of risk/harm/dependence.^35^ Various limitations to different AUD approaches exist, particularly in terms of being unable to fully capture the heterogeneous nature of AUD.^4,35^	AUD concepts are intended for professional purposes but are currently of limited recognition or utility in public discourse.	Despite issues with DSM-5 approach, AUD is recommended as the main term for higher severity/clinical alcohol problems, but with emphasis on further refinement and development.^4,33^
Continuum of alcohol use and harms	A conceptual model focused on the absence of clear categorical boundaries often assumed to exist between groups of ‘problem drinkers’. That is, any level of alcohol use carries risk, all alcohol-related problems exist in degrees of severity.	Continuum beliefs are associated with several positive outcomes vs common binary/disease model concepts.^35,36^ However, continuum models are still relatively under-researched in terms of scientific validity and utility.	Continuum models have been advanced particularly on the basis on public health objectives.^35^ However more nuanced (eg, multi-dimensional) or category-orientated models may be important or necessary for identifying the complex and heterogeneous nature of AUD and responses in a range of contexts.	Advancing a broad continuum model of alcohol use and harms is recommended as a top-level construct for both policy and public understanding. However more complex models (including hierarchical/multi-dimensional models) are required for research and treatment.
Harmful alcohol use (or harmful drinking, or higher risk)	A pattern of alcohol use at which harms are occurring. In the UK, this is defined as more than 35 units per wk for women or 50 units per wk for men.^37^	Harmful use may be an important category of alcohol use for policy makers and other stakeholders to consider, for instance because many harmful drinkers do not identify as having an AUD (or do not formally meet diagnostic criteria) despite experiencing significant harm.^4,38,39,40^	Harmful alcohol use currently has limited recognition or utility in public discourse People drinking at harmful levels are an important group especially since some people with harmful drinking may not meet DSM criteria for AUD.^41,42^	Groups drinking at harmful levels, regardless of whether or not they have a formal diagnosis, are important for stakeholders to consider as they may be less likely to access treatment or support (for instance due to low problem recognition^38^ but still experience significant harm.
Hazardous alcohol use (or increasing risk)	A pattern of alcohol use above lower risk but not reaching harmful levels.	Hazardous alcohol use represents an important level of consumption for public health given the number of people who drink at hazardous levels. This reflects the ‘prevention paradox’, whereby more harm can occur at a population level as a result of hazardous/harmful drinking than more severe problems/AUD.	Hazardous alcohol use currently has limited recognition or utility in public discourse. People drinking at hazardous levels are an important group especially since most people drinking at hazardous levels may not meet DSM criteria for AUD.	Individuals or populations exhibiting hazardous use, regardless of whether or not they have a formal diagnosis, are particularly important for public health/prevention approaches.
Heavy episodic drinking (HED)	The WHO defines heavy episodic drinking as an adult drinking 60 grams or more of pure alcohol on at least one occasion in the past 30 days.^43^	Heavy episodic drinking, similar to ‘binge drinking’ (see Table 2) in public terms, may be useful for describing patterns of drinking that particularly reflect acute harms associated with heavy single occasion amounts.^44^	In most professional contexts, understanding broader patterns of use than HED is likely to be more important for identifying risk and potential interventions/treatment	Heavy episodic drinking is strongly associated with acute harms such as accidents and injury, but frequency and other drinking patterns may also be important to consider.^2,44^
Lived experience (or people with lived/living experience)	People with lived experience may share their experiences of alcohol use and/or AUD for a range of reasons. These insights and perspectives may be utilized in several different ways that may help address use/AUD and associated issues.	Lived experience perspectives can be highly valuable in providing insight and understanding into issues affecting often overlooked or marginalized groups. Lived experience can also be a powerful way to address the stigma of alcohol problems, particularly via humanizing contact^45,46^). Some questions have been raised about what ‘types’ of lived experience of use/AUD are typically shared, and how this may inadvertently perpetrate some stereotypes about use/AUD (eg, as a severe condition requiring abstinence).^47^	Many complex issues arise around how lived experience perspectives are or should be shared in a range of different contexts.^47^ These may relate to important issues concerning different perspectives, stigma and power structures in AUD agendas.^4,9^	A diversification in the current identification of who and what qualifies as lived experience of alcohol problems is important in reducing stigma and increasing understanding of the complex nature and multiple routes to ‘recovery’.^47,48^ ‘People with lived experiences’ (plural) is recommended to emphasize the diversity and multiplicity of lived experience.
Lower risk alcohol use (*Avoid: ‘sensible’, ‘responsible’, ‘safe’, ‘healthy’)	Typically used to refer to drinking within adult recommended guidelines which are deemed to confer a low chance of lifetime alcohol-related health consequences.^49^	Lower risk guidelines are used as a basic public health message to keep the harms associated with alcohol low across the general public. However, alcohol risk guidelines alone have been broadly found to be ineffective at changing behaviour amongst those exceeding them.^50,51^ Some risks such as cancer exist at any level of consumption, thus some groups promote a ‘no safe level’ message,^50^ although the effectiveness of such messages is questioned.^50,52^	Low risk guidelines typically focus on a single weekly consumption level that cannot account for the wide range of factors that influence an individual’s risk. For instance, overall health, age, socio-economic status and many other factors significantly influence individual risk. For AUD groups, reducing alcohol consumption to levels still above lower risk levels may be important and valuable goals and may be considered as non-abstinent recovery.^53^	Low risk guidelines serve to set a broad threshold at which alcohol consumption can be considered ‘low risk’. Guidelines alone are ineffective at reducing alcohol problems. A globally standardized approach to units/standard drinks measurement and associated guidelines may assist with communication efforts.
Alcohol misuse (avoid*)	Alcohol misuse (and sometimes ‘abuse’) is used by some people to describe AUD/drinking patterns/problems	Alcohol misuse may function to describe patterns of alcohol use or behaviours without making inferences about the consequences (eg, suggesting a level of risky consumption without presuming any harms). However, alcohol misuse has been proposed as stigmatizing since ‘misuse’ may feed into notions of personal responsibility and judgement and may be paternalistic.^54^		Alcohol misuse may be best avoided owing to potentially stigmatizing/judgemental connotations.
Prevention	Prevention (ie, public health) approaches to alcohol harms reflect the broad use of alcohol across many populations who may experience risk or harms but not require treatment.	Prevention approaches are of key importance given the extent/cost of harm occurring in populations not meeting AUD criteria (ie, the prevention paradox). Key prevention approaches include pricing, availability and marketing controls, whilst individual-level approaches such as brief intervention can be effective. Prevention approaches however are often politically unpopular and often subject to powerful opposition forces (eg, alcohol industry interests).^55-58^		Prevention/public health approaches are of utmost importance in addressing the overall impact and cost of alcohol problems.
Problem drinking (alcohol problem), or problematic alcohol use (PAU)	Problem drinking/problematic alcohol use (PAU) has been used by some stakeholders as a broad description of harms or risks associated with alcohol use, potentially avoiding issues associated with other terms	Problem drinking/PAU may be useful as a general category of consumption above low risk levels. Until such terms are more universally defined, they may be limited in their utility. Whilst ‘problem’ drinking terminology is not currently thought to be stigmatizing, it may still be interpreted as a false binary of ‘problem’ vs not, thus undermining continuum objectives.^35^		Problem drinking/problematic alcohol use (PAU) may be useful for conveying the broad nature of alcohol-related problems, particularly amongst the public.
Stigma	Stigma is broadly understood as a social process by which people with AUD are labelled, stereotyped and subjected to prejudice and discrimination, or internalize negative strereotypes (self-stigma).	Alcohol problems are heavily stigmatized,^19^ with multiple costs for prevention and recovery.^18^ However, stigma towards people perceived to have alcohol problems is a complex social process with limited research and investment to support evidence-based stigma approaches.^58^	Terms and concepts that may be stigmatizing in some contexts may be de-stigmatizing in others (notably self-labelling within AA).^59,60^	Efforts to understand and reduce alcohol stigma require further prioritization given its impact.
Treatment	Treatment approaches are typically targeted at and accessed by people meeting AUD criteria, especially those with moderate/severe levels.^39,61^	A range of issues relate to alcohol treatment including barriers to funding, accessibility and stigma.	A wide variety of treatment approaches to AUD exist, each of which may rely on or reify terms or concepts which may be problematic in other contexts.	Alcohol treatment forms an important part of efforts to address alcohol problems, although multiple barriers to effective delivery exist including funding, stigma and co-occurring conditions.
Units/standard drinks	A method to identify quantity of ethyl alcohol being consumed (as the psychoactive drug/primary harmful component) in different alcoholic drinks, mainly used to inform recommended drinking guidelines	Worldwide variation exists in how different countries standardize measures for consumption guidelines.		As with recommended guidelines, a globally standardized approach to units/standard drinks measurement and associated guidelines should be sought.

Abbreviations: AUD, alcohol use disorder; UK, United Kingdom; WHO, World Health Organization.

**Table 2.** Key alcohol use and problem terms and concepts in public and recovery contexts.

Term/concept	Summary	Issues and key considerations	Context dependent considerations*	Conclusions and recommendations
Alcoholic (avoid*)	A lay term to describe either another person perceived to have severe alcohol problems, or to self-identify. Alcoholic terminology is typically characterized by loss of control/severe dysfunction as per alcoholism/disease model view/stereotypes.^62^	The term is heavily embedded with negative stereotypes and associated with a range of stigma-related consequences including treatment avoidance. It implies a binary categorization between ‘alcoholics’ and ‘non-problem’ drinkers which can hinder public health goals (eg, problem recognition, non-abstinent recovery).^4^	Some people use alcoholic self-labelling as a way of explicitly recognizing an alcohol problem,^60,63^ or managing shame/guilt as a part of their recovery process.^18,64^ This is particularly the case within Alcoholics Anonymous (AA) which can be an effective peer support group for people with typically more severe AUD.^65^	Alcoholic terminology should be avoided and discouraged except where a person self-labels (eg, within AA).
Alcoholism (avoid*)	Widely used in lay discourse, alcoholism is a historical model of alcohol problems which largely evolved from medical and AA movements that gained significant popularity during the second half of the 20th century.	The term and concept of alcoholism is embedded within stigma and heavily tied to disease model ideas of alcohol problems associated with a range of issues, including very limited scientific/clinical validity and poor prognostic optimism.^4^ As such, it has been replaced by more scientifically derived models which address some of the limitations in capturing the complex and heterogeneous nature of AUD.	Alcoholism is a popular lay concept and may be considered integral to many people’s recovery identity, particularly within the context of AA.^66^ To some extent, disease models may be associated with lower blame as one component of stigma, but are insufficient as a single component to reduce stigma.^18^	Alcoholism is a non-empirically developed concept that should be avoided outside of self-labelling contexts. Stakeholders should use and promote alternative terms and concepts to convey alcohol problems (eg, AUD).
Binge drinking (avoid*)	A mainly lay term describing heavy episodic drinking, although has been defined in policy/professional terms (eg, via either blood alcohol content (BAC) or unit/standard drink thresholds within a single occasion.^41,67^	Binge drinking is a relatively common lay and media term in some countries (eg, the UK). Lay perceptions of binge drinking appear to reflect an intention to achieve a significant degree of intoxication and therefore typically different from consumption-based approaches to defining heavy episodic drinking in professional contexts.^68-70^ Heavy episodic drinking episodes may be a useful indicator of AUD for some groups.		Professionals should seek to avoid using binge drinking or other terminology that may detract from considering overall patterns of use or a broader range of harms than those typically associated with heavy episodic drinking.
Denial (avoid*)	Widely used in lay terms to refer to explicit refusal to accept or identify an alcohol problem.	Denial has been criticized on multiple grounds, particularly in terms of its stigmatizing connotations, and the many complex and often functional or valid reasons behind low problem recognition/acceptance (or refusal to accept an AA-based identity related to powerlessness).^62,71,72^	In some recovery contexts, (notably AA), ‘denial’ may be used to make sense of past experiences or potentially risky thought processes that may lead to return to problematic use.	The term denial should be avoided except for describing one’s own experience. Problem recognition or related constructs (eg, ambivalence, dissonance) should be further used/developed.^73^
Grey area drinking	A term reported to be increasingly used to describe hazardous/harmful levels of consumption amongst people not requiring formal treatment.^4,41^	Grey area drinking is sometimes seen as a lay attempt at reflecting the continuum nature of alcohol use and problems which include those that do not meet more severe popular characterizations of AUD.^35^ This includes increasing recognition and acceptability of non-abstinent recovery and drinking reduction goals.^41,47,74^		Grey area drinking may be a useful lay term/concept to advance recognition of the continuum nature of alcohol use and problems.
Personal responsibility (avoid*)	Personal responsibility is a common public and industry term often used to emphasize the role of individual accountability and choice for drinking behaviours.	Personal responsibility is a pervasive but highly problematic notion in lay understanding of drinking behaviours, primarily because it overlooks the complex and powerful structural and environmental determinants of health.^10,75^		Framings and ideas that relate to notions of personal responsibility to the exclusion of other determinants of use are significant barriers to efforts to address alcohol problems. Stakeholders should work to shift public perceptions to increase understanding of the complex and structural factors that heavily shape drinking behaviours.
Recovery (caution*)	Recovery is defined in a range of different ways, generally seeking to capture the holistic nature of resolution or reduction of harmful substance use/addiction and its consequences as a process.^76-78^	Recovery groups and associated identities are an important part of the resolution of AUD.^60,79,80^ However, ‘recovery’ typically represents a set of ideas that may not capture the broad and heterogeneous nature of alcohol problems.^4^ For instance, people who identify as in recovery typically represent those with more severe AUD experiences and associated alcoholism beliefs such as lifelong abstinence as always necessary.		An expanded understanding of recovery from AUD should be sought in line with efforts to increase recognition of a continuum model of alcohol use and problems.^35^ For instance, recognizing how recovery can include non-abstinent outcomes,^53,81,82^ and can be measured via many other well-being/functioning measures,^74,81,83^ including amongst people with less severe AUD. However ‘recovery’ terminology may not be the best frame to achieve a broadening of understanding of improvements/remission in AUD.
Relapse (avoid*)	A return to problematic alcohol use.	Relapse is used in many different ways, thus lacking precision^84^ and is embedded within ideas of addiction and recovery narratives about alcohol use which may undermine outcomes. For example, the abstinence violation effect^85^ relates to how ‘relapse’ may be worsened by holding beliefs about relapse via a self-fulfilling prophecy effect.^86^	People who self-identify in recovery or are in treatment may commonly use the term relapse. Practitioners should work to help service users to understand the complex nature of alcohol problems and recovery as an ongoing process in which achieving recovery goals is not a linear or single stage process.	Relapse should generally be replaced by return to problematic drinking, or in clinical contexts, less problematic terms such as lapse or setback, or extended into a discussion about identifying learning and future opportunities.

Abbreviations: AA, Alcoholics Anonymous; AUD, alcohol use disorder; UK, United Kingdom.

Our consideration of terms does not take a systematic approach and therefore reflects author-identified terms based on existing literature. Where available, the primary focus was on empirical data in line with the authors’ position that the primary issues presented are best addressed via empirical methodologies. This is not to suggest other methodologies or lived experience accounts are not valuable (and indeed are included), but rather that to evaluate the key implications of terms and concepts as presented is foremost an empirical question. Our selection of terminology involved iterative discussions to reach consensus, with reference to established frameworks such as ICD where relevant. There was limited disagreement between authors but in some instances the first author decided on final wording. In doing so, we acknowledge that there are limitations to the terms and concepts presented and available evidence identified, including the authors’ own interpretations and positionalities. Further, we wish to acknowledge that language and its meanings, including those described here, are never static, but rather in a stage of change and subject to multiple influences, often directly or indirectly in competition with each other. Our results should therefore be interpreted in light of this.

## Terms and Concepts for Alcohol Use and Problems

It is important to note that adopting specific terminologies or constructs such as ‘AUD’ to encompass a myriad of alcohol-related issues raises a number of concerns which are of particular relevance to our objectives. Alcohol use and harms exist as unquestionably complex, heterogenous and dynamic issues, thus we use the term ‘AUD’ only to refer to specific practical and clinical applications within that diagnosis/concept.^33,74^ As identified in Table 1, multiple AUD-related concepts and diagnostic criteria are currently utilized in research, policy and practice—each of which confer strengths and weaknesses. Varied approaches to classifying ‘AUD’ partially reflects historical and socially constructed ideas about alcohol ‘problems’.^87^ Therefore, we use broader, non-diagnostic terms (eg, ‘alcohol problems’) specific to the parameters of alcohol use under consideration to reflect such complexities and avoid reification of ‘AUD’ diagnostic concepts and their limitations. This choice reflects the challenges inherent in setting thresholds which, although required for various research, policy and clinical purposes, raise important issues in terms of how problems are in turn reified by marking who (or which symptoms) does or does not ‘qualify’ as having ‘AUD’.^35,36,88^ Indeed, there are large populations which may not meet AUD criteria but are at risk of or experience a range of alcohol-related problems, and are therefore especially important in terms of prevention efforts (see Table 1) and natural recovery.^4,41^ Classifications including lower risk, hazardous, harmful or heavy episodic drinking (see Table 1) are utilized to identify groups that may not meet AUD criteria, but such approaches are also subject to a range of notable issues and limitations^33,89^ (see Table 1). Our use of the general term ‘alcohol problems’ is therefore used as a limited attempt to mitigate such issues by indicating the broad and heterogeneous range of risks and harms associated with alcohol use. Our broader use of the term ‘alcohol problems’ is therefore intended hold a different meaning from ‘problem drinking’ (see Table 1) which may more typically be associated with a narrower repertoire of problems such as AUD.

We therefore highlight the importance of advancing recognition of a continuum model of alcohol use and problems which emphasizes ‘no clear boundaries’ between groups.^35^ The advantages of a broad continuum model are in part reflected by DSM-5’s (now DSM-5-TR) shift to identifying AUD as either mild, moderate or severe,^90^ although this only goes some way in advancing such objectives.^35^ In some countries including the US, diagnoses from nosologic systems that are categorial in nature are required for alcohol treatment reimbursement. However, it has been proposed that continuum models can be advanced without undermining treatment agendas^36^ and should therefore be promoted amongst the public to advance public health goals, including reducing public perceptions of alcohol problems as confined to more severe ‘dependence’ issues. In turn, continuum beliefs can assist with reducing public stigma whilst increasing problem recognition, particularly amongst populations which may be identified as drinking at ‘hazardous’ or ‘harmful’ levels (see Table 1) levels.^4^

## Not ‘Word Policing’: Evidence for Why Language Matters

Although many organizations have made efforts to attend to potential issues around drug and alcohol-related language and concepts, such actions are not universally supported. For instance, some take the position that language choice is an inconsequential issue, or that calls to attend to language can equate to ‘word policing’ that can provoke resistance and divert attention from more important action^91^ or fail to address the deeper causes of addiction.^92,93^ Another objection made is that of the *euphemism treadmill* in which neutral terms used to replace pejorative ones eventually become pejorative themselves.^94^ However, we argue that the goal of evaluating and shaping alcohol-related terms and concepts must not be considered an end goal in and of itself, but rather an important component of broader, multi-component reform including shifts in public, policy and structural approaches to AUD.^4^ That is, language both reflects and shapes how people understand, feel towards and react to issues of alcohol use and problems, thus indirectly influencing alcohol-related outcomes.

Calls for the use of *person first language* (PFL) have been increasingly endorsed by stigma and substance use researchers.^29,95-97^ PFL relates to the use of terms such as ‘person with an AUD’ in place of stereotype-embedded labels such as ‘addict’ or ‘alcoholic’. PFL thus places emphasis on the person—not the ‘disorder’– in turn reducing implicit and explicit stigma-related judgements and their consequences.^62,98-100^ Other substance-related terminology is also subject to similar attention, particularly terms associated with moralizing or blame-related notions such as ‘abuse’ or ‘misuse’.^54,101^ However, the term *misuse* may be less problematic than *abuse* since the latter is commonly associated with physical or sexual harm, which can imbue harsher internal feelings of shame and judgement, and external discrimination. Instead, *misuse* may be a more neutral term that suggests a harmful or inappropriate behaviour, whilst not explicitly denoting a moral failing. That said, *misuse* may still incur a moral undertone via the implication of individual choice and a failure to exercise control, thus to some extent still potentially a reductionist and stigmatizing framing of substance use.^4,102^

Importantly, ‘word policing’ based objections generally argue that stigma is not removed when stigma-laden terminology is avoided.^91^ We acknowledge such a position as a valid cautionary note. However, PFL and related efforts should not be seen as ‘policing’, but as efforts at guiding empirically beneficial framings, or as leading by example. Although language shift does not *eradicate* stigma or address the causes of alcohol problems, this does not mean that terms and concepts are inconsequential. Indeed, as Corrigan concludes in their caution against ‘word policing’; ‘of course’ stigmatizing terms are ‘not okay’^91^ (p. 235), thus, word change efforts must form part of multi-component strategies to address stigma, including further evaluation of their impact.^91^ We concur; empirical approaches to examine how different terminology and concepts affect key processes or perceptions relating to substance use and problems^59,103,104^ are important because of their more distal *broader* implications.^105,106^

It is therefore important to re-iterate that calls for the adoption of PFL do not necessarily include when people *self-label* via terms such as ‘alcoholic,’ as may be considered an integral aspect to Alcoholics Anonymous (AA) membership.^60^ Over-extending PFL calls to self-labelling contexts would amount to ‘word-policing’, but this is not what is being advocated. Rather, we argue the primary concern regarding alcohol-related language is outside recovery contexts where ‘alcoholism’ terminology and its meanings have skewed public thinking about the nature of alcohol problems.^4^ A growing number of studies have attended to implications of how ‘alcoholism’ concepts or terms may differ from other framings in how the public consider, evaluate and respond to alcohol use and problems. In one study amongst a general population sample, use of the term ‘alcoholic’ was associated with higher stigma rating on both *implicit* (ie, subconscious evaluations) and *explicit* (ie, conscious or reported actions taken based on bias or stigma) measures versus ‘person with an alcohol use disorder’ in an otherwise identical text.^98^ Such findings highlight the clear devaluing consequences of labelling a person as an ‘alcoholic’, which has been argued as inherently stigmatizing due to its deeply embedded negative stereotypes.^62^ That is, the public’s idea of *being* an ‘alcoholic’ casts the person as fundamentally different, diseased and thus belonging to a marked and de-valued out-group.^62,107,108^

Alternatively, a *revaluing* approach argues that rather than adopting PFL, alcoholic/alcoholism terminology itself should be ‘destigmatized’. Although there is little detailed empirical or conceptual work attending to revaluing as a stigma-reduction strategy, it has been proposed as ineffective for more heavily stigmatized terms,^109^ including in the case of ‘alcoholic’ terminology.^62^ That is, where a term is so deeply entrenched in stigma, as is in the case with public stigma towards ‘alcoholism’,^19^ revaluing efforts are unlikely to succeed.^109^ Indeed, it has been argued that the heavy public stigma towards ‘alcoholism’ is in fact driven by many sections of the public who ‘other’ problems in order to protect their own drinking behaviours from stigma.^4^ Thus, whilst we recognize that revaluing has been utilized as a strategy for challenging stigma in some contexts (eg, by some groups to revalue racist or homophobic terms^110^), we propose the extant evidence suggests PFL offers more promise in achieving substance-related stigma-reduction goals.

Further evidence of the problematic effects of ‘alcoholic’ labelling can be seen from a study which found inclusion of the term ‘alcoholic’ was associated with lower problem recognition amongst a community sample of people drinking at harmful levels.^38^ The authors theorized that the stigma-related threat of an ‘alcoholic identity’ resulted in a process of label avoidance, leading to lower evaluations of their level of alcohol-related problems, similar to how people might avoid a ‘mental illness’ label.^111^ Such findings align with broader evidence regarding the effects of the stigma around alcohol problems as a major barrier to treatment engagement^112,113^ and a key predictor or psychological distress and AUD symptoms.^114^ Stigma has also been associated with reduced self-efficacy amongst people who internalize stereotypes via self-stigma.^115^ Clinician stigma towards people seeking or obtaining treatment for substance use results in discrimination and poorer treatment across a range of settings.^116,117^ For example, stigma towards AUD has been identified as a key barrier to the prevention, early detection and intervention of alcohol-associated liver disease.^118^

In contrast, PFL—for the present time at least—avoids embedded negative stereotypes, instead placing emphasis on the *person* rather than reducing them to the ‘disorder’. Since stigma processes are fundamentally dehumanizing, emphasizing the *person* rather than their addiction is a key stigma reduction strategy,^99^ as demonstrated by the effectiveness of *contact* with stigmatized groups.^26,119^ Contact functions to reduce perceptions of fundamental difference (ie, ‘us’ vs ‘them’), which underpin processes of separation and discrimination.^21^ Indeed, ‘othering’ problem drinkers has been proposed as a key driver of the heavy stigma around public perceptions of those deemed to be problem drinkers, particularly by drawing on extreme stereotypes of the ‘alcoholic other’.^18^

## The Role of Context in Evaluating Alcohol Terms and Their Applications

As highlighted, it is essential to consider the importance of *context* when assessing the implications of terminology and concepts for alcohol use and problems. Notably, ‘alcoholic’ self-labelling is a central component of AA, in part reflecting member’s commitment to overcoming ‘denial’, identifying with other group members,^60^ and for some, resolving guilt and shame (facets of self-stigma).^93,64^ However, many people who disengage from AA reportedly do so because they struggle with ‘alcoholic’ self-labelling and/or the corresponding disease-based characterization of their experiences.^120^ People are therefore aware of and evaluate the harmful stigma consequences specifically associated with ‘alcoholic’ labelling.^38,98,113^ However, adopting a stigmatizing identity may be managed via a range of self-stigma ‘coping responses’.^121^ These coping strategies have the potential to be helpful or harmful to the individual and their recovery, thus termed the ‘paradox of self-stigma’.^122^ For some, actively fighting stigma may become of source of self-worth and part of a recovery identity^123^ and marker of group membership.^60^ However, for others, AUD self-stigma via alcoholic self-labelling may undermine recovery through reduced self-efficacy,^115^ increased feelings of guilt and shame^124^ or reduced autonomy and recovery optimism.^62,125,126^ This may reflect how the accounts of people who self-label as ‘alcoholics’ often convey mixed views about the implications for the self and management of self-stigma.^93,64^

This is not to argue that people should not self-label as ‘alcoholics’, particularly given its centrality to AA membership as an often effective and free route to abstinence-based recovery.^65^ However, it is notable that AA members are cautious and strategic about disclosing an alcoholic identity outside of AA and are aware others will view and treat them differently.^127^ Research suggests that some AA members report wishing to distance themselves from an alcoholic identity at later stages in their recovery.^128^ It should therefore be noted that whilst alcoholic self-labelling may itself become a positive aspect of a recovery identity, this experience is far from universal and potential negative consequences should not be overlooked. As such, the potential for alcoholic labelling to help or hinder efforts to address a person’s AUD ultimately depends on how the person themselves evaluate the term and its implications for the self. However, we argue the most important consideration here is that most people with AUD will never consider themselves as ‘alcoholics’ or engage with AA.^47,74,129,130^ This reflects the many ways in which dominant public beliefs about ‘alcoholism’ overlook the broad continuum nature of alcohol use and problems, and why othering one’s self from an ‘alcoholic’ identity appears a common strategy for resisting problem recognition.^38^ Consequently, there is a need for alternative and less stigma-embedded terms and concepts for people to evaluate their drinking against.^4^

## Complexity in Concepts for Alcohol Use and Problems

Although the implications of specific terms are more easily tested via experimental studies, *concepts* attempting to capture alcohol use and problems are by their nature more complicated to evaluate. AUD is the main contemporary conceptualization for alcohol problems in professional contexts, yet still reflects a varied set of classification approaches and limitations, which are largely divergent from the public’s beliefs about what alcohol problems exist as.^4,87^ Notably, representations of ‘alcoholism’ as a chronic relapsing disorder/disease are still evident in many scientific articles,^131^ including as a ‘chronically relapsing brain disease’ according to the National Institute on Alcohol Abuse and Alcoholism (NIAAA).^132^ However, disease-orientated concepts have been highlighted as problematic for several reasons.^62,133^ These include important challenges to the validity of AUD/addiction as a primarily biomedical or disease entity,^133,134^ but also a tension with ‘upstream’ public health efforts which emphasize population level approaches.^4^ Indeed, sections of the alcohol industry have been known to favour disease model framings in order to undermine support for population level measures and protect profits.^75,135^ In this context, a disease framing is used to denote alcohol problems as an issue of a biologically distinct minority population. In turn, industry narratives portray the majority of the drinking population as ‘in control’ via an emphasis on ‘personal responsibility’, thus discrediting the role of public health policies to control price, availability or marketing.^10,136^

Nonetheless, disease models may reduce some components of stigma, notably *blame*,^137,138^ or at least in specific contexts.^59,118^ This may reflect how addiction-related beliefs may serve as *functional attributions*.^139,140^ Attributions are thus mechanisms for how terms or concepts are deployed to achieve objectives such as sense-making or recovery processes, or goals such as stigmatization or policy influence (by either health or industry groups). However, rises in disease model attributions amongst the public have failed to reduce public stigma towards both mental health problems and alcohol problems.^141,142^ One explanation is that the public still holds moralizing views towards people with addiction, even when not considering them as blameworthy.^143^ This may derive from evolutionary motives such as disease avoidance or efforts to signal or punish social norm contraventions, often reflecting emotionally mediated stigma responses such as fear or anger.^15,144^ Notably, in one recent experimental study of public stigma towards people with alcohol problems, psychological (ie, coping mechanism) and nature (ie, innate drive to alter consciousness) models of AUD were associated with lower stigma, but no difference was found for a disease model.^145^

Disease orientated models of alcohol problems may therefore be ‘mixed blessings’^146,147^—potentially valuable in some recovery or medical contexts,^118^ but also indirectly facilitating public stigma^62^ whilst obstructing the uptake of more effective alternative models for advancing public health goals.^4,145^ Several alternative models have been proposed as potentially advantageous to public health goals including stigma reduction. These include promoting the continuum nature of alcohol use and problems which emphasize the absence of categorical differences between groups^35^ and increase recognition of drinking reduction goals as a valid route to recovery.^148^ Psychological models typically emphasize the development of alcohol problems in response to lived experiences or psychological distress.^145,149,150^ Dynamic models of responsibility have proposed that with increasing severity of problems, increased recognition of the role of society, rather than only the individual, is more conducive to recovery outcomes and stigma reduction.^118^ This approach is consistent with efforts to emphasize distinctions between *responsibility* (whereby individuals are still accountable for their actions) and stigmatizing *blame*,^151^ and arguments against the use of social ‘disapproval’ to address addiction problems across society.^144^ Other objectives proposed as important to reducing alcohol-related problems include better recognizing the role of quality-of-life measures and drinking reduction goals.^74,76,152^

Without delving further into the complex arguments about the precise nature of alcohol problems and associated concepts, we aim to highlight that its framing is embedded within social, commercial and political contexts which are often overlooked.^87,153,154^ As such, alcohol-related terminology and concepts are inescapably embedded within—and shape—agendas including research funding,^155^ policy making/lobbying and regulation,^10,12^ and ‘recovery’ movements.^156^

## The Need for Clarity and Precision in Research, Policy and Practice

Precision in terminology is crucial throughout the research journey, starting from conceptualization and study design through to communication and implementation.^157^ This includes how alcohol problems or risks are communicated, with ongoing challenges identified related to how ‘lower risk’ drinking guidelines are communicated,^50,158^ including efforts to enable people to identify or monitor their consumption via ‘units’ or ‘standard drinks’ (see Table 1). Similarly, terms such as ‘binge drinking’ (see Table 2) have been identified as problematic due to significant variation in their meaning and application.^67,159^ Notably, alcohol-related terminology within academic research journals is frequently inconsistent, imprecise or stigmatizing. For example, as described, ‘alcoholism’ is generally a highly problematic term, yet is often used to describe AUD or alcohol dependence criteria, sometimes without any clarification.^160^ Shi et al^97^ identified prevalent use of the term ‘alcoholic’ in alcohol research over the last decade. Specifically, in 2020, over 40% of articles searched for included ‘alcoholic’, whilst other studies have identified the persistence of other stigmatizing terminology within publications of AUD clinical trials.^161^ Stigmatizing terminology (ie, ‘alcoholic’, ‘alcoholism’, ‘alcohol abuse’) is also widespread within clinical contexts including on websites of addiction and liver disease providers.^162^ Imprecision or inaccuracy is also commonly evident in describing differing levels of alcohol use.^41^ For instance, levels of ‘hazardous’ use (eg, as per ICD-11) may be incorrectly described as ‘harmful’,^160^ thus, conflating different categories and undermining the advantages of emphasizing the heterogeneous nature of alcohol use and problems.^35^ A wide range of other terms are also used as imprecise primary descriptors of AUD criteria or related components in contemporary scientific publications including ‘excessive’^163^ or ‘unhealthy’ use,^164^ ‘abuse’,^165^ ‘misuse’, ‘addiction’^166^ and ‘relapse’.^167^

One important opportunity to improve scientific and professional conceptualizations and discourse is via addiction ontologies. Ontologies are designed to provide accessible and consistent definitions and concepts to provide clarity to complex constructs and thus reduce ambiguity.^157^ The Addiction Ontology (AddictO) provides a transparent guide for using key terms to ensure that the writer’s definition is clear, whilst facilitating conceptual understanding of important but complex related concepts,^168^ though it does not explicitly advocate for PFL. Additionally, researchers and clinicians must endeavour to use clinically accurate and medically precise language when discussing AUD symptomology (ie, physiological dependence, craving), or frequency and patterns of consumption.

Precision in terminology reflects the importance of delivering treatment (see Table 1) in a consistent and appropriate way to ensure and assess the best possible outcomes for individuals and providers. For example, a common, lay conception of the term ‘relapse’ implies moral failure if a person resumes alcohol consumption after a period of omittance, and should be strictly avoided in the context of treatment provision.^169^ Conversely, a systematic review found that the term has been used with multiple different connotations within published AUD research,^84^ though often without clear definitions or empirical criteria. In the same context, researchers should avoid using euphemistic phrases such as ‘suffers from’ or ‘has problems with’ which infer a subjective helplessness to the person or group under study. Instead, authors should objectively state the symptoms the participant(s) or group is reported to be experiencing. In addition to the *Addiction Ontology*, other examples include an online ‘addictionary’ (https://www.recoveryanswers.org/addiction-ary/), and the College on Problems of Drug Dependence has published a list of resources to provide guidance in destigmatizing language in research (https://cpdd.org/destigmatizing-sud-language/).

## Key Terms and Concepts for Advancing the Reduction of Alcohol Problems

We have discussed a number of terms and concepts as of key relevance to advancing efforts to reduce alcohol problems, although many other important terms identified in Tables 1 and 2 have not been discussed in detail. Since we did not take a systematic approach to included literature, we recognize this as an important limitation and suggest that future research systematically develop and evaluate alcohol-related terms and constructs and their likely impact on efforts to reduce alcohol harms. Despite these limitations, we argue the key priority for evaluating terms and concepts for alcohol use and problems reflects the many issues associated with alcoholism models and associated stereotypes. For example, *denial* (see Table 2) is another common term that has been cautioned against due to a lack of scientific validity and stigmatizing implications. Notably, ‘denial’ leads to over-simplification of the many complex reasons behind people not explicitly self-identifying as having a ‘problem’.^170^ As Pickard^71^ examines, there are various context-related factors that undermine the legitimacy of ‘denial’ stereotypes, such as the absence of clear harms, the subjectivity of what ‘problems’ are, or significant variation in norms about what constitutes ‘addiction’ and ‘denial’. Morris et al^170^ propose alcohol problem recognition involves a range of social (eg, stigma-related threats) and cognitive (eg, implicit bias) processes which are overlooked when ‘denial’ terminology is used.

Relatedly, the roles of *natural recovery* (ie, self-change) and *non-abstinent recovery* (historically termed ‘controlled drinking’) also remain overlooked or discredited despite clear evidence for their existence as important routes to recovery, perhaps because of the dominant societal emphasis on ‘alcoholism’ models of AUD.^47,74,76,152,171,172^ Although abstinence is an important and valid goal for many people with AUD, a 2020 systematic review and meta-analysis found no inferiority in outcomes for those with non-abstinent goals.^171^ Despite this, cynicism towards non-abstinent recovery persists, reflecting a history of events that have undermined and even actively smeared controlled drinking research.^173^ One proposed response is an increased availability of lived experience narratives that highlight drinking reductions as legitimate and effective responses to alcohol problems for many.^47^ A focus on outcomes other than alcohol use, notably quality of life measures, has also been advocated for improving the uptake and effectiveness of alcohol treatment.^74,83,174,175^

‘Natural recovery’ broadly denotes the resolution of alcohol problems without formal help or treatment, but is also overlooked as an important facet of alcohol use and problems.^176^ Indeed, longitudinal studies show most people who develop alcohol problems resolve these without formal treatment,^177^ in turn underpinning calls for broader recognition of the multiple and complex pathways to resolving alcohol problems.^74^ Cynicism towards natural recovery also reflects the pathologization of alcohol problems as a ‘disease’ in need of treatment (see Table 1), thus undermining problem recognition and self-change intentions amongst people with lower severity problems such as those with ‘hazardous’ levels of consumption or ‘mild’ or sub-threshold AUD.^41,62^ Lay terms such as *grey area drinking* (see Table 2) reflect public attempts to capture such experiences of alcohol problems without using ‘alcoholism’ orientated terms and concepts^129,156^ and therefore warrant further investigation for their utility in advancing the reduction of alcohol problems.^4^

## Conclusion

Language and concepts relating to alcohol use and problems are complex, dynamic and subject to multiple contextual issues. This in turn reflects a range of challenges in how concepts such as AUD and associated facets are understood and addressed across professional and lay settings. Nonetheless, some important objectives are apparent. Most notably, outside of recovery contexts, PFL should be adopted wherever possible, and professional stakeholders should cease use of ‘alcoholic’ and ‘alcoholism’ terminology due to the clear problems associated with their use, including their over-application in wider discourse relating to alcohol use and problems. In doing so, it is important to emphasize that the use of PFL reflects empirical evidence pertaining to how alcohol use and problems are understood and treated in order to mitigate ‘word-policing’ and related concerns. Crucially, seeking to progress alcohol-related language and concepts must not be seen as an end in itself, rather as a means to facilitate important but more distal alcohol-related outcomes including public attitudes and policy decisions. Specific objectives include developing more nuanced public understandings about the nature of alcohol use and problems as a broad continuum, and recognition of the multiple routes to its resolution, including via drinking reduction goals and self-change. Indeed, the voices and direct contributions of people with lived experience are crucial to many such objectives, but their inclusion must reflect consideration of the broad heterogeneous nature of alcohol problems and the under-representation of its existence in less severe forms, and experiences of non-abstinent ‘recovery’. A further important shift should be to move away from individually focused ideas about alcohol problems as primarily biomedical problems or failures of ‘personal responsibility’, instead towards increased recognition of the broader structural and environmental factors at play.

## References

[1] World Health Organization. Global Status Report on Alcohol and Health 2018. World Health Organization; 2018.

[2] Rehm J Badaras R Ferreira-Borges C , et al. Impact of the WHO ‘best buys’ for alcohol policy on consumption and health in the Baltic countries and Poland 2000-2020. Lancet Reg Heal Eur. 2023;33:100704. 10.1016/j.lanepe.2023.100704 PMC10636269 37953993

[3] McCambridge J Lesch M. Are we moving into a new era for alcohol policy globally? An analysis of the Global Alcohol Action Plan 2022-30. BMJ Glob Health. 2024;9:14246. 10.1136/bmjgh-2023-014246 PMC10895216 38388164

[4] Morris J Boness CL Burton R. (Mis)understanding alcohol use disorder: making the case for a public health first approach. Drug Alcohol Depend. 2023;253:111019. 37952353 10.1016/j.drugalcdep.2023.111019 PMC11061885

[5] Entman RM. Framing: toward clarification of a fractured paradigm. J Commun. 1993;43:51-58.

[bibr6-29768357241312555] Halliday MA Webster JJ , eds. On Language and Linguistics. Continuum; 2003.

[bibr7-29768357241312555] Patton MQ. Overview: language matters. New Dir Eval. 2000;2000:5-16.

[8] Blankenship KL Craig TY. Language use and persuasion: multiple roles for linguistic styles. Soc Personal Psychol Compass. 2011;5:194-205.

[9] Room R. Stigma, social inequality and alcohol and drug use. Drug Alcohol Rev. 2005;24:143-155. 16076584 10.1080/09595230500102434

[bibr10-29768357241312555] Maani Hessari N Petticrew M . What does the alcohol industry mean by ‘Responsible drinking’? A comparative analysis. J Public Heal (United Kingdom). 2018;40:90-97. 10.1093/pubmed/fdx040 28398571

[bibr11-29768357241312555] Hawkins B Holden C. Framing the alcohol policy debate: industry actors and the regulation of the UK beverage alcohol market. Crit Policy Stud. 2013;7:53-71.

[12] Fitzgerald N Angus K Howell R , et al. Changing public perceptions of alcohol, alcohol harms and alcohol policies: a mixed-methods study to develop novel framing approaches. Addiction. 2024. 10.1111/add.16743 PMC11907333 39711155

[13] Taylor S. Moving beyond the other: a critique of the reductionist drugs discourse. Tijdschr Cult Crim. 2016;6:100-118.

[bibr14-29768357241312555] Stevens A. ‘Being human’ and the ‘moral sidestep’ in drug policy: explaining government inaction on opioid-related deaths in the UK. Addict Behav. 2019;90:444-450. 30220439 10.1016/j.addbeh.2018.08.036

[15] Oaten M Stevenson RJ Occhipinti S Tapp C Case TI. The factorial structure of stigma and its targets. Soc Psychol (Gott). 2022;53:96-106.

[16] Van Amsterdam J Opperhuizen A Koeter M Van Den Brink W . Ranking the harm of alcohol, tobacco and illicit drugs for the individual and the population. Eur Addict Res. 2010;16:202-207. 20606445 10.1159/000317249

[17] Burton R Henn C Lavoie D , et al. A rapid evidence review of the effectiveness and cost-effectiveness of alcohol control policies: an English perspective. Lancet (London, England). 2017;389:1558-1580. 27919442 10.1016/S0140-6736(16)32420-5

[18] Morris J Schomerus G. Why stigma matters in addressing alcohol harm. Drug Alcohol Rev. 2023;42:1264-1268. 37042726 10.1111/dar.13660

[19] Kilian C Manthey J Carr S , et al. Stigmatization of people with alcohol use disorders: an updated systematic review of population studies. Alcohol Clin Exp Res. 2021;45:899-911. 33970504 10.1111/acer.14598

[20] Pescosolido BA Martin JK. The stigma complex. Annu Rev Sociol. 2015;41:87-116. 26855471 10.1146/annurev-soc-071312-145702 PMC4737963

[bibr21-29768357241312555] Link BG Phelan JC. Conceptualizing stigma. Annu Rev Sociol. 2001;27:363-385.

[22] Stangl AL Earnshaw VA Logie CH , et al. The health stigma and discrimination framework: a global, crosscutting framework to inform research, intervention development, and policy on health-related stigmas. BMC Med. 2019;17:1-13. 30764826 10.1186/s12916-019-1271-3 PMC6376797

[23] Carter MJ. The hermeneutics of frames and framing: an examination of the media’s construction of reality. SAGE Open. 2013;3:1-12.

[bibr24-29768357241312555] Gross R . The Psychology of Prejudice. L. Erlbaum Associates; 2020.

[bibr25-29768357241312555] Law J . Collateral realities. In: Baert P Rubio FD , eds. The Politics of Knowledge. Routledge; 2013:156-178.

[26] Corrigan PW Schomerus G Shuman V , et al. Developing a research agenda for reducing the stigma of addictions, part II: lessons from the mental health stigma literature. Am J Addict. 2016;26:67-74. 27875626 10.1111/ajad.12436

[27] National Institute on Drug Abuse (NIDA). Words matter: preferred language for talking about addiction. 2021. Accessed June 15, 2023. https://nida.nih.gov/research-topics/addiction-science/words-matter-preferred-language-talking-about-addiction

[28] Stuart GL Ramsey SE. What’s in a name? Destigmatizing language regarding people who use alcohol or drugs in publications and journal title. Subst Use. 2024;18:11782218231222908 38433750 10.1177/11782218231222908 PMC10906496

[29] Broyles LM Binswanger IA Jenkins JA , et al. Confronting inadvertent stigma and pejorative language in addiction scholarship: a recognition and response. Subst Abus. 2014;35:217-221. 24911031 10.1080/08897077.2014.930372 PMC6042508

[30] Bathish R Madden A Duff C Ritter A. Guiding principles for breaking down drug-related stigma in academic writing. Int J Drug Policy. 2024;131:104515. 39208549 10.1016/j.drugpo.2024.104515

[31] Humphreys K Calder R Marsden J Day E. How addiction handles disagreements over potentially harmful terminology. Addiction. 2023;118:1833-1834. 37489005 10.1111/add.16302

[32] COMMITTEE SHHSASC. NJ Legislature. 2023. Accessed January 23, 2024. https://www.njleg.state.nj.us/bill-search/2022/S3511

[33] Boness CL Watts AL Moeller KN Sher KJ. The etiologic, theory-based, ontogenetic hierarchical framework of alcohol use disorder: a translational systematic review of reviews. Psychol Bull. 2021;147:1075-1123. 35295672 10.1037/bul0000333 PMC8923643

[34] NICE. Alcohol-Use Disorders: Diagnosis, Assessment and Management of Harmful Drinking and Alcohol Dependence [CG115]. The British Psychological Society and The Royal College of Psychiatrists; 2011. Accessed August 31, 2016. https://www.nice.org.uk/guidance/cg115/evidence 22624177

[35] Morris J Boness CL Witkiewitz K. Should we promote alcohol problems as a continuum? Implications for policy and practice. Drugs Educ Prev Policy. 2023;31:271-281. 10.1080/09687637.2023.2187681 PMC11052541 38682086

[36] Morris J Boness CL Witkiewitz K. A continuum model of alcohol use and problems can advance public health goals without undermining treatment agendas. Reply to commentaries. Drugs (Abingdon Engl). 2024;31:287-288. 38863692 10.1080/09687637.2023.2244658 PMC11164551

[37] Department of Health. Alcohol Guidelines Review: Report from the Guidelines Development Group to the UK Chief Medical Officers; 2016. Accessed April 2, 2018. https://www.gov.uk/government/consultations/health-risks-from-alcohol-new-guidelines

[38] Morris J Moss AC Albery IP Heather N. The ‘alcoholic other’: harmful drinkers resist problem recognition to manage identity threat. Addict Behav. 2022;124:107093. 34500234 10.1016/j.addbeh.2021.107093

[39] Dunne J Kimergård A Brown J , et al. Attempts to reduce alcohol intake and treatment needs among people with probable alcohol dependence in England: a general population survey. Addiction. 2018;113:1430-1438. 29575560 10.1111/add.14221

[40] Khadjesari Z Stevenson F Toner P Linke S Milward J Murray E. ‘I’m not a real boozer’: a qualitative study of primary care patients’ views on drinking and its consequences. J Public Health (Bangkok). 2018;41:185-191. 10.1093/pubmed/fdy067 29912419

[41] Volpicelli JR Menzies P. Rethinking unhealthy alcohol use in the United States: a structured review. Subst Abus Res Treat. 2022;16:11782218221111832. 10.1177/11782218221111832 PMC9310219 35899221

[42] Rehm J Marmet S Anderson P , et al. Defining substance use disorders: do we really need more than heavy use? Alcohol Alcohol. 2013;48:633-640. 23926213 10.1093/alcalc/agt127

[43] Alcohol Research: Current Reviews Editorial Staff. Drinking patterns and their definitions. Alcohol Res. 2018;39:17-18. 30557143 10.35946/arcr.v39.1.03 PMC6104961

[44] Rehm J Gmel GE Gmel G , et al. The relationship between different dimensions of alcohol use and the burden of disease—an update. Addiction. 2017;112:968-1001. 28220587 10.1111/add.13757 PMC5434904

[45] Hamilton LJ Coleman ME Krendl AC. Contact reduces substance use stigma through bad character attributions, especially for U.S. health care professionals. Psychol Addict Behav. 2023;37:734-745. 37668564 10.1037/adb0000953

[46] Pescosolido BA Manago B . Getting underneath the power of ‘contact’: revisiting the fundamental lever of stigma as a social network phenomenon. In: Major B Dovidio JF Link BG , eds. The Oxford Handbook of Stigma, Discrimination, and Health. Vol. 1. Oxford University Press; 2017:397-411.

[47] Morris J Cox S Moss AC Reavey P . Drinkers like us? The availability of relatable drinking reduction narratives for people with alcohol use disorders. Addict Res Theory. 2022:1-8.

[48] Tucker JA Witkiewitz K . Historical and contemporary perspectives on pathways to recovery from alcohol use disorder. In: Witkiewitz K, Tucker JA, eds. Dynamic Pathways to Recovery from Alcohol Use Disorder. Cambridge University Press; 2021:3-22.

[49] Bellis M Jones L . CMO alcohol guidelines review—a summary of the evidence of the health and social impacts of alcohol consumption; 2016. Accessed April 2, 2018. http://www.cph.org.uk/publication/cmo-alcohol-guidelines-review-a-summary-of-the-evidence-of-the-health-and-social-impacts-of-alcohol-consumption/

[50] Ferri M Mouteney J Griffiths P. Low-risk drinking guidelines: a pragmatic approach to health promotion? Addiction. 2019;114:605-607. 30659671 10.1111/add.14532

[51] Holmes J Beard E Brown J , et al. Effects on alcohol consumption of announcing and implementing revised UK low-risk drinking guidelines: findings from an interrupted time series analysis. J Epidemiol Community Heal. 2020;74:942-949. 10.1136/jech-2020-213820 PMC7576577 32684524

[52] Morris J Tattan-Birch H Albery IP , et al. Look away now! Defensive processing and unrealistic optimism by level of alcohol consumption. Psychol Health. 2024:1-19. 10.1080/08870446.2024.2316681 38379336

[53] Votaw VR Witkiewitz K . The clinical benefits of non-abstinent outcomes in alcohol use disorder treatment: evidence from clinical trials and treatment implications. In: Mueller S, Heilig M, eds. Alcohol and Alcohol-Related Diseases. 2023:341-364.

[54] Watts AL Boness CL. A proposal to revise the title of HiTOP’s substance abuse subconstruct. OSF Prepr. Published online 2022. doi:10.17605/OSF.IO/ZMSCU

[55] McCambridge J Kypri K Drummond C , et al. Alcohol harm reduction: corporate capture of a key concept. PLoS Med. 2014;11:e1001767. 10.1371/journal.pmed.1001767 PMC4260782 25490717

[bibr56-29768357241312555] Mitchell G Lesch M McCambridge J. Alcohol industry involvement in the moderate alcohol and cardiovascular health trial. Am J Public Health. 2020;110:485-488. 32078349 10.2105/AJPH.2019.305508 PMC7067094

[bibr57-29768357241312555] Maani N Schalkwyk MC van, Wiseman A Petticrew M. Commercially driven efforts to frame alcohol harms have no place in UK health policy development. BMJ. 2024;385:q800. 10.1136/bmj.q800 38575194

[58] Krendl AC Perry BL. Stigma toward substance dependence: causes, consequences, and potential interventions. Psychol Sci Public Interest. 2023;24:90-126. 37883667 10.1177/15291006231198193

[59] Kelly JF Greene MC Abry A. A US national randomized study to guide how best to reduce stigma when describing drug-related impairment in practice and policy. Addiction. 2021;116:1757-1767. 33197084 10.1111/add.15333 PMC8124079

[60] Glassman HS Moensted M Rhodes P Buus N. The politics of belonging in alcoholics anonymous: a qualitative interview study. Am J Community Psychol. 2022;70:33-44. 34761820 10.1002/ajcp.12568

[61] Freyer J Coder B Bischof G , et al. Intention to utilize formal help in a sample with alcohol problems: a prospective study. Drug Alcohol Depend. 2007;87:210-216. 16979304 10.1016/j.drugalcdep.2006.08.018

[62] Morris J . Before ‘rock bottom’? Problem framing effects on stigma and change among harmful drinkers. In: Heather N Field M Moss AC Satel S , eds. Evaluating the Brain Disease Model of Addiction. Routledge; 2022:187-195.

[63] Humphreys K. Community narratives and personal stories in alcoholics anonymous. J Community Psychol. 2000;28:495-506.

[64] Hill JV Leeming D. Reconstructing ‘the alcoholic’: recovering from alcohol addiction and the stigma this entails. Int J Ment Health Addict. 2014;12:759-771.

[65] Kelly JF Abry A Ferri M Humphreys K. Alcoholics anonymous and 12-step facilitation treatments for alcohol use disorder: a distillation of a 2020 cochrane review for clinicians and policy makers. Alcohol Alcohol. 2020;55:641-651. 32628263 10.1093/alcalc/agaa050 PMC8060988

[66] Glassman HS Moensted ML Rhodes P , et al. Obvious benefits but hidden costs: a critical exploration of the impact of adopting the ‘master narrative’ in Alcoholics Anonymous. J Subst Use Addict Treat. 2023;148:209010. 36931603 10.1016/j.josat.2023.209010

[67] Herring R Berridge V Thom B. Binge drinking: an exploration of a confused concept. J Epidemiol Community Health. 2008;62:476-479. 18477743 10.1136/jech.2006.056721

[68] Lyons AC Emslie C Hunt K . Staying ‘in the zone’ but not passing the ‘point of no return’: embodiment, gender and drinking in mid-life. In: Cohn S, ed. From Health Behaviours to Health Practices: Critical Perspectives. Vol. 36. Wiley/Blackwell; 2014:106-119. 10.1111/1467-9566.12103 PMC4211357 24447057

[bibr69-29768357241312555] Stevely AK Holmes J Meier PS. Contextual characteristics of adults’ drinking occasions and their association with levels of alcohol consumption and acute alcohol-related harm: a mapping review. Addiction. 2020;115:218-229. 31655026 10.1111/add.14839

[70] Davies EL Cooke R Visser RO de, Conroy D. Calling time on responsible drinking: a qualitative study of perceptions of information on alcohol product labels. Br J Health Psychol. 2023;28:320-337. 36263853 10.1111/bjhp.12627

[71] Pickard H. Denial in addiction. Mind Lang. 2016;31:277-299.

[72] Dare PAS Derigne L. Denial in alcohol and other drug use disorders: a critique of theory. Addict Res Theory. 2010;18:181-193.

[73] Morris J Richards DK Albery IP. Problem recognition as a discrete concept for change processes in problematic alcohol use. Curr Addict Reports. 2025. 10.1007/s40429-025-00634-x PMC11839834 39989883

[74] Witkiewitz K Tucker JA . Dynamic pathways to recovery from alcohol use disorder. In: Tucker JA Witkiewitz K , eds. Dynamic Pathways to Recovery from Alcohol Use Disorder. Cambridge University Press; 2021:415-426.

[75] McCambridge J Garry J Room R. The origins and purposes of alcohol industry social aspects organizations: insights from the tobacco industry documents. J Stud Alcohol Drugs. 2021;82:740-751. 34762033

[76] Witkiewitz K Montes KS Schwebel FJ Tucker JA. What is recovery? Alcohol Res. 2020;40:01. 10.35946/arcr.v40.3.01 PMC7505137 32983748

[bibr77-29768357241312555] Ray LA Lim AC Shoptaw S . What defines a clinically meaningful outcome in the treatment of substance use disorders: ‘getting your life back.’ Addiction 2019;114:18-20. 30426617 10.1111/add.14455

[78] Kelly JF Magill M Stout RL. How do people recover from alcohol dependence? A systematic review of the research on mechanisms of behavior change in Alcoholics Anonymous. Addict Res Theory. 2009;17:236-259.

[79] Best D Beckwith M Haslam C , et al. Overcoming alcohol and other drug addiction as a process of social identity transition: the social identity model of recovery (SIMOR). Addict Res Theory. 2016;24:111-123.

[80] Best D Sondhi A Patton D , et al. A cluster analysis of European life in recovery data: what are the typical patterns of recovery experience? Drugs Educ Prev Policy. 2024;32(1):1-9.

[81] Witkiewitz K. ‘Success’ following alcohol treatment: moving beyond abstinence. Alcohol Clin Exp Res. 2013;37(Suppl. 1):E9-E13. 10.1111/acer.12001 23075307

[82] Cunningham JA Schell C Walker H Godinho A. Patterns of remission from alcohol dependence in the United Kingdom: results from an online panel general population survey. Subst Abus Treat Prev Policy. 2024;19:1-6. 10.1186/s13011-023-00588-1 PMC10768276 38178169

[83] Witkiewitz K Tucker JA . Whole person recovery from substance use disorder: a call for research examining a dynamic behavioral ecological model of contexts supportive of recovery. Addict Res Theory. 2024;33(1):1-12. 40059906 10.1080/16066359.2024.2329580 PMC11883499

[84] Maisto SA Witkiewitz K Moskal D Wilson AD. Is the construct of relapse heuristic, and does it advance alcohol use disorder clinical practice? J Stud Alcohol Drugs. 2016;77:849. 27797685 10.15288/jsad.2016.77.849 PMC5088167

[85] Heather N Winton A Rollnick S. An empirical test of ‘a cultural delusion of alcoholics’. Psychol Rep. 1982;50:379-382. 7089126 10.2466/pr0.1982.50.2.379

[86] Miller WR Westerberg VS Harris RJ , et al. What predicts relapse? Prospective testing of antecedent models. Addiction. 1996;91(Suppl.):S155-S172. 8997790

[87] Boness CL Votaw VR Francis MW Watts A. Alcohol use disorder conceptualizations and diagnoses reflect their sociopolitical context. Addict Res Theory. 2022;31:1-6. 10.1080/16066359.2022.2150935 PMC10656047 37981984

[88] Boness CL Lane SP Sher KJ. Not all alcohol use disorder criteria are equally severe: toward severity grading of individual criteria in college drinkers. Psychol Addict Behav. 2019;33:35-49. 30676037 10.1037/adb0000443 PMC6494471

[89] Saunders JB Degenhardt L Reed GM Poznyak V. Alcohol use disorders in ICD-11: past, present, and future. Alcohol Clin Exp Res. 2019;43:1617-1631. 31194891 10.1111/acer.14128

[90] American Psychiatric Association. Diagnostic and Statistical Manual of Mental Disorders. American Psychiatric Association; 2013.

[91] Corrigan PW. Beware the word police. Psychiatr Serv. 2019;70:234-236. 30717642 10.1176/appi.ps.201800369

[92] Alexander BK . Replacing the BDMA: a paradigm shift in the field of addiction. In: Heather N, Field M, Moss AC, Satel S, eds. Evaluating the Brain Disease Model of Addiction. Routledge; 2022:522-538.

[93] Hassett-Walker C. Recovering individuals’ feelings about addict and alcoholic as stigmatized terms: implications for treatment. Subst Abus Res Treat. 2023;17:11782218231213769. 10.1177/11782218231213769 PMC10687927 38033430

[94] Pinker S. The Blank Slate: The Modern Denial of Human Nature. Penguin Books Ltd; 2003.

[95] Robinson SM. ‘Alcoholic’ or ‘person with alcohol use disorder’? Applying person-first diagnostic terminology in the clinical domain. Subst Abus. 2017;38:9-14. 27925860 10.1080/08897077.2016.1268239

[bibr96-29768357241312555] Hartwell M Naberhaus B Arnhart C , et al. The use of person-centered language in scientific research articles focusing on alcohol use disorder. Drug Alcohol Depend. 2020;216:108209. 32801060 10.1016/j.drugalcdep.2020.108209

[97] Shi HD McKee SA Cosgrove KP. Why language matters in alcohol research: reducing stigma. Alcohol Clin Exp Res. 2022;46:1103-1109. 35727299 10.1111/acer.14840 PMC9246863

[98] Ashford RD Brown AM Curtis B. Substance use, recovery, and linguistics: the impact of word choice on explicit and implicit bias. Drug Alcohol Depend. 2018;189:131-138. 29913324 10.1016/j.drugalcdep.2018.05.005 PMC6330014

[bibr99-29768357241312555] McGinty EE Barry CL. Stigma reduction to combat the addiction crisis—developing an evidence base. N Engl J Med. 2020;382:1291-1292. 32242352 10.1056/NEJMp2000227

[100] Wakeman SE . The language of stigma and addiction. In: Avery JD Avery JJ , eds. The Stigma of Addiction: An Essential Guide. Springer International Publishing; 2019:71-80.

[101] Kelly JF Westerhoff CM. Does it matter how we refer to individuals with substance-related conditions? A randomized study of two commonly used terms. Int J Drug Policy. 2010;21:202-207. 20005692 10.1016/j.drugpo.2009.10.010

[102] Acuff SF Strickland JC Smith K Field M. Heterogeneity in choice models of addiction: the role of context. Psychopharmacology (Berl). 2024;241:1753-1769. 10.1007/s00213-024-06646-1 38990313

[103] Pennington CR Monk RL Heim D Rose A Gough T. The labels and models used to describe problematic substance use impact discrete elements of stigma: a registered report. Psychol Addict Behav. 2023;37:723-733. 37166945 10.1037/adb0000919

[104] Kelly JF Dow SJ Westerhoff C. Does our choice of substance-related terms influence perceptions of treatment need? An empirical investigation with two commonly used terms. J Drug Issues. 2010;40:805-818.

[105] Wogen J Restrepo MT. Human rights, stigma, and substance use. Health Hum Rights. 2020;22:51. 32669788 PMC7348456

[106] Earnshaw VA. Stigma and substance use disorders: a clinical, research, and advocacy agenda. Am Psychol. 2020;75:1300-1311. 33382299 10.1037/amp0000744 PMC8168446

[107] Piras AP Preti A Moro MF , et al. Does calling alcoholism an illness make a difference? The public image of alcoholism in Italy. Drug Alcohol Depend. 2016;166:39-44. 27373185 10.1016/j.drugalcdep.2016.06.015

[108] Young LB. Joe sixpack: normality, deviance, and the disease model of alcoholism. Cult Psychol. 2011;17:378-397.

[109] Bolinger RJ . The language of mental illness. In: Khoo J Sterken R , eds. The Routledge Handbook of Social and Political Philosophy of Language. Routledge; 2021:389-404.

[110] Wang CS Whitson JA Anicich EM Kray LJ Galinsky AD. Challenge your stigma: how to reframe and revalue negative stereotypes and slurs. Curr Dir Psychol Sci. 2017;26:75-80.

[111] Thoits PA. ‘I’m not mentally ill’: identity deflection as a form of stigma resistance. J Health Soc Behav. 2016;57:135-151. 27284073 10.1177/0022146516641164

[112] May C Nielsen AS Bilberg R. Barriers to treatment for alcohol dependence. J Drug Alcohol Res. 2019;8:1-17.

[113] Glass JE Mowbray OP Link BG Kristjansson SD Bucholz KK. Alcohol stigma and persistence of alcohol and other psychiatric disorders: a modified labeling theory approach. Drug Alcohol Depend. 2013;133:685. 24071569 10.1016/j.drugalcdep.2013.08.016 PMC3980578

[114] Cruwys T Selwyn J Rathbone JA Frings D. Discrimination and social identity processes predict impairment and dysfunction among heavy drinkers. Soc Sci Med. 2024;343:116549. 38219413 10.1016/j.socscimed.2023.116549

[115] Schomerus G Corrigan PW Klauer T Kuwert P Freyberger HJ Lucht M. Self-stigma in alcohol dependence: consequences for drinking-refusal self-efficacy. Drug Alcohol Depend. 2011;114:12-17. 20933344 10.1016/j.drugalcdep.2010.08.013

[116] Carpenter JE Catalanotti J Notis M , et al. Use of nonstigmatizing language is associated with improved outcomes in hospitalized people who inject drugs. J Hosp Med. 2023;18:670-676. 37286190 10.1002/jhm.13146 PMC10524912

[117] Buchman DZ Imahori D Lo C , et al. The influence of using novel predictive technologies on judgments of stigma, empathy, and compassion among healthcare professionals. AJOB Neurosci. 2024;15:32-45. 37450417 10.1080/21507740.2023.2225470

[118] Schomerus G Leonhard A Manthey J , et al. The stigma of alcohol-related liver disease and its impact on healthcare. J Hepatol. 2022;77:516-524. 35526787 10.1016/j.jhep.2022.04.026

[119] Brown R Hewstone M. An integrative theory of intergroup contact. Adv Exp Soc Psychol. 2005;37:255-343.

[120] Glassman HS Rhodes P Buus N. Exiting alcoholics anonymous disappointed: a qualitative analysis of the experiences of ex-members of AA. Heal (United Kingdom). 2022;26:411-430. 10.1177/1363459320961438 32993383

[121] Corrigan PW Rao D. On the self-stigma of mental illness: stages, disclosure, and strategies for change. Can J Psychiatry. 2012;57:464-469. 22854028 10.1177/070674371205700804 PMC3610943

[122] Corrigan PW Watson AC. The paradox of self-stigma and mental illness. Clin Psychol Sci Pract. 2002;9:35-53.

[123] Jetten J Haslam SA Cruwys T Branscombe NR . Social identity, stigma, and health. In: Major B Dovidio JF Link BG , eds. The Oxford Handbook of Stigma, Discrimination, and Health. Vol. 1. Oxford University Press; 2017:301-316.

[124] Léna M Tristan H Isabelle V. Guilt in alcohol use: a systematic review. Addict Behav. 2023;137:107531. 36332517 10.1016/j.addbeh.2022.107531

[125] Dar-Nimrod I Zuckerman M Duberstein PR. The effects of learning about one’s own genetic susceptibility to alcoholism: a randomized experiment. Genet Med. 2013;15:132-138. 22935722 10.1038/gim.2012.111

[126] Lindgren KP Burnette JL Hoyt CL Peterson KP Neighbors C. Growth mindsets of alcoholism buffer against deleterious effects of drinking identity on problem drinking over time. Alcohol Clin Exp Res. 2020;44:233-243. 31709565 10.1111/acer.14237 PMC6980889

[127] Romo LK Dinsmore DR Watterson TC. ‘Coming out’ as an alcoholic: how former problem drinkers negotiate disclosure of their nondrinking identity. Health Commun. 2016;31:336-345. 26360915 10.1080/10410236.2014.954090

[128] Howard J. Expecting and accepting: the temporal ambiguity of recovery identities. Soc Psychol Q. 2006;69:307-324.

[129] Khadjesari Z Stevenson F Godfrey C Murray E. Negotiating the ‘grey area between normal social drinking and being a smelly tramp’: a qualitative study of people searching for help online to reduce their drinking. Heal Expect. 2015;18:2011-2020. 10.1111/hex.12351 PMC5016787 25676536

[130] Walters GD. Twelve reasons why we need to find alternatives to alcoholics anonymous. Addict Disord Their Treat. 2002;1:53-59.

[131] Cunningham JA McCambridge J. Is alcohol dependence best viewed as a chronic relapsing disorder? Addiction. 2012;107:6-12. 21981681 10.1111/j.1360-0443.2011.03583.x PMC3272223

[132] NIAAA. Alcohol use disorder or #AUD is a chronic relapsing #brain disease marked by compulsive alcohol use, loss of control over alcohol intake, and a negative emotional state when not drinking. 2019. Accessed August 7, 2023. https://twitter.com/NIAAAnews/status/1102977687814094850

[133] Heather N Field M (Matthew) Moss AC Satel SL , eds. Evaluating the Brain Disease Model of Addiction. Routledge; 2022.

[134] Lewis M. Brain change in addiction as learning, not disease. N Engl J Med. 2018;379:1551-1560. 30332573 10.1056/NEJMra1602872

[135] Bhattacharya A Angus C Pryce R Holmes J Brennan A Meier PS. How dependent is the alcohol industry on heavy drinking in England? Addiction. 2018;113:2225-2232. 30136436 10.1111/add.14386

[136] Maani N Ci van Schalkwyk M Petticrew M. Under the influence: system-level effects of alcohol industry-funded health information organizations. Health Promot Int. 2023;38:1-9. 10.1093/heapro/daad167 PMC10721437 38097395

[137] Schomerus G Matschinger H Angermeyer MC. Continuum beliefs and stigmatizing attitudes towards persons with schizophrenia, depression and alcohol dependence. Psychiatry Res. 2013;209:665-669. 23465293 10.1016/j.psychres.2013.02.006

[138] Schomerus G Matschinger H Angermeyer MC. Causal beliefs of the public and social acceptance of persons with mental illness: a comparative analysis of schizophrenia, depression and alcohol dependence. Psychol Med. 2014;44:303-314. 23574735 10.1017/S003329171300072X

[139] Pennington CR Monk RL Heim D , et al. The labels and models used to describe problematic substance use impact discrete elements of stigma: a registered report. Psychol Addict Behav. 2023;37:723-733. 37166945 10.1037/adb0000919

[140] Monk RL Heim D. Self-image bias in drug use attributions. Psychol Addict Behav. 2011;25:645-651. 21988477 10.1037/a0025685

[141] Schomerus G Lucht M Holzinger A Matschinger H Carta MG Angermeyer MC. The stigma of alcohol dependence compared with other mental disorders: a review of population studies. Alcohol Alcohol. 2011;46:105-112. 21169612 10.1093/alcalc/agq089

[142] Pescosolido BA Martin JK Long JS , et al. ‘A disease like any other’? A decade of change in public reactions to schizophrenia, depression, and alcohol dependence. Am J Psychiatry. 2010;167:1321-1330. 20843872 10.1176/appi.ajp.2010.09121743 PMC4429867

[143] Frank LE Nagel SK. Addiction and moralization: the role of the underlying model of addiction. Neuroethics. 2017;10:129-139. 28725284 10.1007/s12152-017-9307-x PMC5486499

[144] Morris J Kummetat JL Schomerus G. Can ‘justified disapproval’ be separated from addiction stigma? An empirical focus is required. Addict Res Theory. Published online 2024. doi:10.1080/16066359.2024.2394424

[145] Rundle SM Cunningham JA Hendershot CS. Implications of addiction diagnosis and addiction beliefs for public stigma: a cross-national experimental study. Drug Alcohol Rev. 2021;40:842-846. 33493359 10.1111/dar.13244

[146] Kvaale EP Haslam N Gottdiener WH. The ‘side effects’ of medicalization: a meta-analytic review of how biogenetic explanations affect stigma. Clin Psychol Rev. 2013;33:782-794. 23831861 10.1016/j.cpr.2013.06.002

[147] Lebowitz MS Appelbaum PS. Beneficial and detrimental effects of genetic explanations for addiction. Int J Soc Psychiatry. 2017;63:717-723. 29058960 10.1177/0020764017737573 PMC5693609

[148] Leonhard A Leonhard C Sander C Schomerus G. The effect of alcohol use disorder symptom and recovery narratives on problem-recognition: a randomized online trial. Addict Behav. 2022;134:107426. 35870440 10.1016/j.addbeh.2022.107426

[149] Weine ER Kim NS Lincoln AK. Understanding lay assessments of alcohol use disorder: need for treatment and associated stigma. Alcohol Alcohol. 2016;51:98-105. 26113491 10.1093/alcalc/agv069 PMC4678947

[150] Wiens TK Walker LJ. The chronic disease concept of addiction: helpful or harmful? Addict Res Theory. 2015;23:309-321.

[151] Pickard H. Responsibility without blame for Addiction. Neuroethics. 2017;10:169-180. 28725286 10.1007/s12152-016-9295-2 PMC5486507

[152] Witkiewitz K Morris J Tucker JA . Commentary on Henssler et al.: the public health case for promoting and valuing drinking reductions in the treatment of alcohol use disorder. Addiction. 2021;116:1988-1989. 33554367 10.1111/add.15429

[153] Morris J Boness CL Burton R Dar-Nimrod I Moss A. [Commentary] Balancing the bio in a biopsychosocial model of hazardous drinking and alcohol use disorders. Qeios. Published online April 7, 2023. doi:10.32388/I1120F

[154] Room R. The World Health Organization and alcohol control. Addiction. 1984;79:85-92. 10.1111/j.1360-0443.1984.tb00250.x 6584165

[155] Midanik LT. Biomedicalization and alcohol studies: implications for policy. J Public Health Policy. 2004;25:211-228. 15255386 10.1057/palgrave.jphp.3190021

[156] Atkinson AM Meadows BR Nicholls E Sumnall HR. ‘Some days I am a lunatic that thinks I can moderate’: amalgamating recovery and neo-liberal discourses within accounts of non-drinking among women active in the ‘positive sobriety’ community on Instagram in the UK. Int J Drug Policy. 2023;112:103937. 36566608 10.1016/j.drugpo.2022.103937

[157] Hastings J Cox S West R Notley C. Addiction ontology: applying basic formal ontology in the addiction domain. Qeios. Published online December 7, 2020. doi:10.32388/HZHJIP

[158] Holmes J Sumnall H. On risk in addiction science, policy and debate. Addiction. 2017;112:1895-1897. 28872721 10.1111/add.13962

[159] Conroy D Griffin C Morton C. Defending, contesting and rejecting formal drinker categories: how UK university students identify as ‘light-drinkers’ or ‘non-drinkers’. Drugs Educ Prev Policy. 2022;29:509-518.

[160] Yoshimoto H Kawaida K Dobashi S Saito G Owaki Y. Effect of provision of non-alcoholic beverages on alcohol consumption: a randomized controlled study. BMC Med. 2023;21:379. 37784187 10.1186/s12916-023-03085-1 PMC10544561

[161] Hartwell M Lin V Hester M , et al. Stigmatizing terminology for outcomes and processes (STOP) in alcohol research: a meta-epidemiologic assessment of language used in clinical trial publications. J Addict Med. 2022;16:527-533. 35120059 10.1097/ADM.0000000000000960 PMC9531923

[162] Mahle R Okanlawon A Luther J Louissaint J Zhang W. Stigmatizing language for alcohol use disorder and liver disease on liver transplant center websites. JAMA Netw Open. 2024;7:e2355320. 10.1001/jamanetworkopen.2023.55320 PMC10853823 38329758

[163] Crowley R Hilden D Beachy M ; Health and Public Policy Committee of the American College of Physicians. Excessive alcohol use and alcohol use disorders: a policy brief of the American College of Physicians. Ann Intern Med. 2024;177:656-657. 38648644 10.7326/M23-3434

[164] Rose GL Badger GJ Skelly JM MacLean CD Ferraro TA Helzer JE. A randomized controlled trial of brief intervention by interactive voice response. Alcohol Alcohol. 2017;52:335-343. 28069598 10.1093/alcalc/agw102 PMC5397878

[165] Ray LA Hutchison KE Leventhal AM , et al. Diagnosing alcohol abuse in alcohol dependent individuals: diagnostic and clinical implications. Addict Behav. 2009;34:587-592. 19362427 10.1016/j.addbeh.2009.03.028 PMC3741094

[166] Curyło M Czerw A Rynkiewicz-Andryśkiewicz M , et al. Measuring the intensity of stress experienced and its impact on life in patients with diagnosed alcohol use disorder. J Clin Med 2024;13:572. 38276078 10.3390/jcm13020572 PMC10816737

[167] Maisto SA Roos CR Hallgren KA Moskal D Wilson AD Witkiewitz K. Do alcohol relapse episodes during treatment predict long-term outcomes?: Investigating the validity of existing definitions of alcohol use disorder relapse. Alcohol Clin Exp Res. 2016;40:2180. 10.1111/acer.13173 PMC5048537 27591560

[168] Notley C West R Soar K Hastings J Cox S. Toward an ontology of identity-related constructs in addiction, with examples from nicotine and tobacco research. Addiction. 2023;118:548-557. 36370069 10.1111/add.16079

[169] Sliedrecht W Roozen H De Waart R Dom G Witkiewitz K. Variety in alcohol use disorder relapse definitions: should the term ‘relapse’ be abandoned? J Stud Alcohol Drugs. 2022;83:248-259. 35254248

[170] Morris J Albery IP Moss AC Heather N . Promoting problem recognition amongst harmful drinkers: a conceptual model for problem framing factors. In: Frings D Albery IP , eds. The Handbook of Alcohol Use. Elsevier; 2021:221-236.

[171] Henssler J Müller M Carreira H Bschor T Heinz A Baethge C. Controlled drinking—non-abstinent versus abstinent treatment goals in alcohol use disorder: a systematic review, meta-analysis and meta-regression. Addiction. 2021;116:1973-1987. 33188563 10.1111/add.15329

[172] Chew J Morris J Bartlett G Frings D. Evaluation of web-based digital intervention to change individual’s drinking behaviours. Alcohol Treat Q. 2024;42:1-19.

[173] Sobell MB Sobell LC. Controlled drinking after 25 years: how important was the great debate? Addiction. 1995;90:1149-1153; discussion 1157-1177. 7580815

[174] Witkiewitz K Tucker JA. Abstinence not required: expanding the definition of recovery from alcohol use disorder. Alcohol Clin Exp Res. 2020;44:36-40. 31709568 10.1111/acer.14235 PMC6980896

[175] Haeny AM Schick MR Crouch MC , et al. Self-change from alcohol problems among racially and ethnically minoritized adults: a systematic review. Curr Addict Rep. 2024;11:818-837. 10.1007/s40429-024-00591-x PMC13229543 42238810

[176] Klingemann H Glass JE . Natural recovery from alcohol problems. In: Heather N Peters T Stockwell T , eds. International Handbook of Alcohol Dependence and Problems. John Wiley & Sons, Ltd; 2001:649-662.

[177] Dawson DA Grant BF Stinson FS Chou PS Huang B Ruan WJ. Recovery from DSM-IV alcohol dependence: United States, 2001-2002. Addiction. 2005;100:281-292. 15733237 10.1111/j.1360-0443.2004.00964.x

